# Beyond BMI: Metabolic Signatures of Obstructive Coronary Artery Disease in a Sudanese Cohort

**DOI:** 10.3390/jcm14238345

**Published:** 2025-11-24

**Authors:** Ramaze F. Elhakeem, Mohamed F. Lutfi, Abdelkarim A. Abdrabo, Ahmed B. Ali, Mohamed Y. Sukkar

**Affiliations:** 1 Department of Physiology, College of Medicine, Qassim University, Buraydah 51452, Saudi Arabia; ra.morsi@qu.edu.sa; 2 College of Applied Medical Science, Buraydah Colleges, Buraydah 51418, Saudi Arabia; abdrabokarim@bpc.edu.sa; 3 Faculty of Medicine, Al Neelain University, Khartoum 12217, Sudan; draali99@gmail.com; 4 Nile College of Medicine, Khartoum 11111, Sudan; profmys@gmail.com

**Keywords:** body mass index, coronary artery disease, insulin resistance, lipid profile, Sudan

## Abstract

**Background:** CAD continues to be a major global cause of morbidity and mortality, but its metabolic underpinnings in African populations remain poorly characterized. Conventional predictors such as body mass index (BMI) and insulin resistance markers may not fully capture risk in these settings. **Methods:** We conducted a hospital-based cross-sectional, hypothesis-generating study of 138 Sudanese patients with symptomatic angina who underwent elective coronary angiography at El-Shaab Teaching Hospital, Khartoum, Sudan. Clinical and demographic data were collected, and venous blood samples were analyzed for fasting blood glucose (FBG), fasting insulin (FI), and lipid profiles. Insulin sensitivity was estimated using the Quantitative Insulin Sensitivity Check Index (QUICKI). Patients were categorized into obstructive CAD (≥50% stenosis, n = 72) and non-obstructive CAD (<50% stenosis, n = 66) groups. Statistical analyses included group comparisons, BMI-stratified analyses, and logistic regression modeling. **Results:** Patients with obstructive CAD were significantly older (*p* = 0.044), predominantly male (80.6% vs. 50.0%, *p* < 0.001), and more frequently diabetic (*p* = 0.011). BMI was unexpectedly lower in the obstructive group (*p* = 0.044). FBG was significantly higher and HDL-C lower in the obstructive group, both before and after adjusting for BMI, while FI and QUICKI did not differ significantly between groups. Logistic regression identified age, male gender, and FBG as independent predictors of obstructive CAD. **Conclusions:** Elevated fasting glucose and reduced HDL-C, rather than BMI or classical insulin resistance indices, appear to be key metabolic signatures of obstructive CAD in Sudanese patients. These findings underscore the importance of population-specific risk markers to improve early detection and tailored prevention strategies in underrepresented African cohorts.

## 1. Introduction

Coronary artery disease (CAD) continues to represent a significant global health challenge, largely attributed to metabolic and lifestyle factors [1]. Among these, insulin resistance and dyslipidemia have emerged as critical contributors to atherogenesis [2,3,4,5]. Despite extensive research in high-income countries, the burden and pathophysiological mechanisms of CAD in low- and middle-income settings, particularly in sub-Saharan Africa, remain inadequately characterized [5,6].

Insulin resistance, often preceding the clinical onset of type 2 diabetes mellitus, contributes to endothelial dysfunction, low-grade, and atherogenic lipid alterations [1,7,8,9,10]. Surrogate markers such as fasting insulin (FI) and the quantitative insulin sensitivity check index (QUICKI) are widely used to estimate insulin sensitivity in epidemiological studies [11,12]. Although their predictive validity is well-supported in Western populations, few studies have explored their utility in African or Middle Eastern cohorts undergoing coronary angiography [13,14].

In addition to insulin dynamics, lipid abnormalities, especially low HDL cholesterol (HDL-C) and elevated LDL-C/HDL-C ratios, serve as established cardiovascular risk factors [8,15,16]. However, the relationship between lipid parameters and body composition, particularly in non-obese patients, remains complex and may vary with ethnicity, metabolic phenotype, and disease stage [4,9,17,18,19,20,21,22,23].

African populations remain significantly under-represented in metabolic and cardiovascular research, limiting the generalizability of established insulin-resistance indices and lipid-based markers to diverse genetic, nutritional, and environmental contexts such as Sudan [24]. Moreover, cardiometabolic diseases are rising rapidly across Sub-Saharan Africa alongside urbanization and lifestyle change, yet data linking metabolic dysfunction to confirmed coronary lesions are scarce [25]. Insulin resistance is a central mechanism in atherosclerosis, yet its predictive value in non-Western cohorts remains uncertain [26]. Recent metabolomics work shows that amino-acid, lipid, and fatty-acid pathways are perturbed in both CAD and metabolic syndrome, underscoring the potential of low-cost metabolic biomarkers to enhance risk stratification beyond anthropometry [27].

This study explores the metabolic signatures underlying CAD in a Sudanese cohort undergoing elective coronary angiography (ECA). While body mass index (BMI) is widely used in risk prediction, it fails to capture hidden metabolic dysfunctions in many individuals [28]. By assessing insulin resistance markers and lipid profiles across BMI categories, this study aims to go beyond traditional anthropometric measures. In regions like Sudan, where cardiometabolic diseases are rising and resources are limited, low-cost biomarkers could transform early risk detection. Our findings may help redefine cardiovascular risk assessment in non-obese, high-risk individuals and guide early detection strategies and tailored prevention efforts in populations that are often underrepresented in global cardiovascular research.

## 2. Materials and Methods

This hospital-based cross-sectional study was designed to investigate the relationship between insulin resistance markers, lipid profile parameters, and coronary angiographic findings in patients undergoing ECA. The research was conducted at the cardiac catheterization units of El-Shaab Teaching Hospital, Khartoum, Sudan.

Ethical approval for this study was granted by the Ethics Review Committee of the Faculty of Medicine, University of Khartoum, Sudan (approval code: UKFM/072021). Institutional permissions were secured from the hospital administration. Each participant provided written informed consent after receiving a full explanation of the study protocol. The investigations were conducted in accordance with the principles outlined in the Declaration of Helsinki [29].

Consecutive recruitment was used to minimize selection bias; every eligible patient presenting during the study period was invited to participate. A total of 138 adult patients with clinically suspected stable angina, referred for ECA, were enrolled. Eligibility criteria included symptoms suggestive of myocardial ischemia warranting further evaluation through coronary imaging. Referral for angiography followed cardiologist assessment based on typical angina symptoms together with supportive non-invasive indicators of ischemia, including electrocardiographic changes, cardiac biomarker findings, and, when available, stress-test results. Patients included in the study were adults aged ≥18 years who were referred for elective diagnostic angiography. Patients previously diagnosed with structural cardiac abnormalities unrelated to coronary artery disease, such as cardiomyopathy, congenital heart disorders, or rheumatic valvular lesions, were excluded from participation.

All participants were instructed by their managing cardiologists to report in a fasting state on the morning of their scheduled angiography. Following informed consent, a structured data collection sheet was used to document clinical history and perform a physical examination. Venous blood samples (5 mL) were obtained from each patient. Approximately 2 mL of the sample was transferred into fluoride oxalate tubes for fasting glucose analysis. The remaining sample was allowed to clot and centrifuged to isolate serum for biochemical investigations.

Fasting blood glucose (FBG) and lipid profile components (total cholesterol, triglycerides, high-density lipoprotein cholesterol (HDL-C), and low-density lipoprotein cholesterol (LDL-C)) were quantified with standard enzymatic techniques on an automated biochemistry analyzer (Shenzhen Mindray Bio-Medical Electronics, Shenzhen, China).

FI levels were measured using the radioimmunoassay (RIA) technique, employing a SOURCERER gamma counter (OAKFIELD Company, London, UK) along with dedicated insulin assay kits (China Institute for Atomic Energy, Beijing, China). QUICKI was computed using the formula: QUICKI = 1/[log (FI µU/mL) + log (fasting glucose mg/dL)]. A QUICKI value ≤ 0.30 was considered indicative of insulin resistance.

BMI was determined by dividing a participant’s weight in kilograms by the square of their height in meters. Mean arterial blood pressure (MABP) was estimated using the standard formula: MABP = diastolic pressure + 1/3 (systolic − diastolic blood pressures).

Coronary angiograms were interpreted by experienced cardiologists blinded to laboratory findings. Based on angiographic results, patients were stratified into two groups: those with obstructive coronary artery disease (OCAD), defined as ≥50% luminal narrowing in one or more major coronary arteries (n = 72) [30,31,32,33], and those without significant obstruction (non-obstructive CAD, NOCAD; n = 66). The study was not designed to compare CAD versus non-CAD patients but to elucidate metabolic differences across disease severity.

### Statistical Analysis

**Figure 1:**
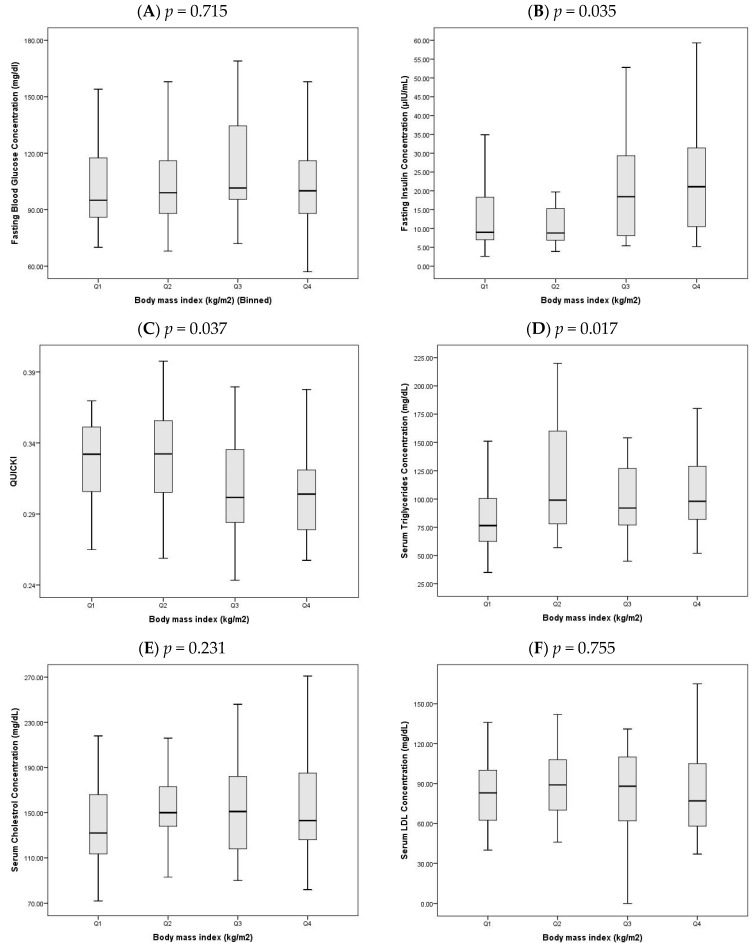

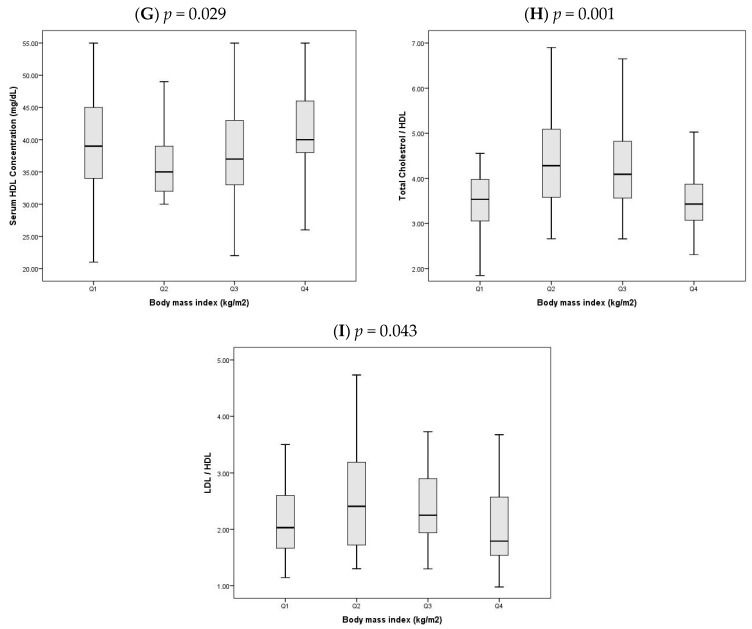
Distribution of glycemic and lipid profile measurements among the BMI Quartiles. Median (Q1–Q3) values of (**A**) FBG (mg/dL), (**B**) FI (μIU/mL), (**C**) QUICKI, (**D**) Triglycerides (mg/dL), (**E**) Total Cholesterol (mg/dL), (**F**) LDL-C (mg/dL), (**G**) HDL-C (mg/dL), (**H**) Cholesterol/HDL-C ratio, and (**I**) LDL-C/HDL-C ratio. *p* values indicate statistically significant differences in glycemic and lipid profile measures across BMI quartiles, based on the Kruskal–Wallis test.

All statistical analyses were conducted using IBM SPSS Statistics version 21 (IBM Corp., Armonk, NY, USA). Normality of continuous variables was evaluated with the Shapiro–Wilk test. Variables with a normal distribution are expressed as mean ± standard deviation (SD), whereas non-normally distributed data are reported as median with interquartile ranges (Q1–Q3). Categorical variables are summarized as frequencies and percentages. Group comparisons between OCAD and NOCAD patients were performed using the independent samples *t*-test for normally distributed variables, the Mann–Whitney U test for skewed data, and the Chi-square test for categorical variables. Given the statistically significant difference in BMI between the OCAD and NOCAD groups, the study population was further stratified into BMI quartile groups. This stratification enabled a more detailed examination of how glycemic and lipid profile measurements varied across increasing levels of BMI. Specifically, the following biomarkers were compared across BMI quartiles: FBG, FI, QUICKI, triglycerides, total cholesterol, LDL-C, HDL-C, cholesterol/HDL-C, and LDL-C/HDL-C ratios. Since many of these variables were not normally distributed, comparisons between quartiles were made using the Kruskal–Wallis test. The results are presented in Figure 1, which details the distribution of metabolic markers across the four BMI quartile groups. Given the exploratory nature of this cross-sectional study, *p*-values were not adjusted for multiple testing; all results should therefore be interpreted as hypothesis-generating.

To determine independent predictors of OCAD, a binary logistic regression analysis was conducted using a forward stepwise (likelihood ratio) method. Candidate variables included age, gender, BMI, MABP, FBG, FI, QUICKI, lipid profile components, and medical history of hypertension, diabetes mellitus, and smoking. The adequacy of the model was evaluated using the Hosmer–Lemeshow goodness-of-fit test, while the Nagelkerke R^2^ value was used to estimate the proportion of variance explained. Odds ratios (ORs) with corresponding 95% confidence intervals (CIs) were computed for each predictor. Statistical significance was defined as a *p*-value < 0.05.

## 3. Results

A total of 138 patients undergoing coronary angiography were included in the analysis, with 72 patients classified as OCAD and 66 as having NOCAD. As shown in Table 1, patients in the OCAD group were significantly older than those in the NOCAD group (*p* = 0.044). The proportion of males was notably higher in the OCAD group (80.6%) compared to the NOCAD group (50.0%) (*p* < 0.001). Interestingly, BMI was lower in the OCAD group (*p* = 0.044), whereas no significant differences were observed between the groups in MABP (*p* = 0.504), smoking status (*p* = 0.196), or past history of hypertension (*p* = 0.835). Diabetes mellitus was significantly more common in the OCAD group (*p* = 0.011).

Table 2 presents the distribution of glycemic and lipid parameters between OCAD and NOCAD groups, both unadjusted and adjusted for BMI. FBG levels were significantly higher in the OCAD group before (*p* = 0.017) and after BMI adjustment (*p* = 0.026). FI levels were lower in the OCAD group, though the difference only demonstrated a trend toward significance (*p* = 0.055). The QUICKI did not differ significantly between groups (*p* = 0.800). HDL-C cholesterol levels were significantly lower in the OCAD group in both unadjusted (*p* = 0.006) and BMI-adjusted analyses (*p* = 0.037). No significant group differences were found for triglycerides, total cholesterol, LDL-C cholesterol, or lipid ratios (cholesterol/HDL-C, LDL-C/HDL-C), either before or after adjusting for BMI.

In light of the significant difference in BMI between OCAD and NOCAD groups, the study population was stratified into quartiles based on BMI to explore trends in glycemic and lipid parameters (Figure 1A–I). All values are reported as median (Q1–Q3) and compared using the Kruskal–Wallis test.

A. FBG did not differ significantly across quartiles (*p* = 0.715, Figure 1A), with median values ranging from 101.0 (86.5–151.5) in Q1 to 105.5 (90.0–127.0) in Q4.
B. FI levels increased significantly with higher BMI (*p* = 0.035, Figure 1B), rising from 9.7 (7.2–22.6) in Q1 to 19.6 (11.8–31.4) in Q4, indicating increased insulin secretion in individuals with greater adiposity.
C. QUICKI decreased significantly across quartiles (*p* = 0.037, Figure 1C), from 0.33 (0.30–0.35) in Q1 to 0.30 (0.28–0.32) in Q4, reflecting a decline in insulin sensitivity with increasing BMI.
D. Triglyceride levels also showed a significant upward trend (*p* = 0.017, Figure 1D), increasing from 76.5 (62.5–100.5) in Q1 to 102.5 (82.0–132.0) in Q4.
E. Total cholesterol did not show a significant difference between quartiles (*p* = 0.231, Figure 1E), with values ranging from 132.0 (113.5–166.0) in Q1 to 141.0 (126.0–190.5) in Q4.
F. LDL-C cholesterol remained statistically unchanged across quartiles (*p* = 0.755, Figure 1F), with medians of 83.0 (62.5–100.0) in Q1 and 78.5 (59.0–108.5) in Q4.
G. HDL-C cholesterol varied significantly (*p* = 0.029, Figure 1G), with the lowest median in Q2 (35.0 (32.0–38.5)) and the highest in Q4 (40.0 (37.0–46.5)).
H. The cholesterol/HDL-C ratio differed significantly across quartiles (*p* = 0.001, Figure 1H), peaking in Q2 at 4.3 (3.6–5.1) and decreasing in Q4 to 3.4 (3.1–4.1).
I. Similarly, the LDL-C/HDL-C ratio varied significantly (*p* = 0.043, Figure 1I), highest in Q2 at 2.4 (1.8–3.1) and lowest in Q4 at 1.8 (1.6–2.6).

A binary logistic regression analysis was performed to identify independent predictors of OCAD (Table 3). The final model included four variables: age, gender, FBG, and FI. The overall model was statistically significant (Chi-square = 31.62, df = 4, *p* < 0.001), indicating that the combination of these predictors reliably distinguished between patients with and without OCAD. The Nagelkerke R^2^ value was 0.341, meaning the model explained approximately 34.1% of the variance in the likelihood of having obstructive CAD. Model calibration was assessed using the Hosmer–Lemeshow goodness-of-fit test, which was non-significant (*p* = 0.114), confirming that the model fit the observed data well. Additionally, the model achieved a classification accuracy of 72.9%, suggesting good discriminatory ability between OCAD and non-OCAD cases. Older age (*p* = 0.003), male gender (*p* = 0.006), and higher fasting glucose (*p* = 0.013) were significant independent predictors of OCAD. FI was negatively associated with OCAD but did not reach statistical significance (*p* = 0.082).

Post-angiography management, including medical therapy optimization and revascularization decisions, followed institutional protocols and were comparable between groups; these data were not within the analytic scope of the present study.

## 4. Discussion

The findings of this study offer important insights into the metabolic and demographic characteristics associated with CAD in patients undergoing ECA. To better emphasize the clinical relevance of these results, the discussion is organized into key thematic domains, each addressed as a subsection: demographic risk factors for OCAD, glycemic status and insulin dynamics, BMI and metabolic complexity, BMI-stratified analysis of metabolic markers, predictors of OCAD in regression analysis, and study appraisal, implications, and future directions. Each of these components contributes uniquely to the cardiovascular risk profile and helps clarify the distinctions between patients with OCAD and those with NOCAD.

### 4.1. Demographic Risk Factors for OCAD

The present study identified significant age and gender differences between OCAD and NOCAD patients. Patients with OCAD were notably older, consistent with the well-established understanding that advancing age contributes to cumulative exposure to atherogenic factors [1,33,34]. This finding aligns with large-scale studies like the INTERHEART global case–control study, which highlighted an age-associated increase in myocardial infarction risk, particularly in males [35].

The proportion of NOCAD (48%) observed in this cohort is consistent with contemporary evidence indicating that a substantial percentage of symptomatic patients undergoing coronary evaluation exhibit non-obstructive lesions. Prior studies report that approximately 30–60% of individuals referred for coronary angiography for angina symptoms show NOCAD [36] and that up to half of symptomatic patients may demonstrate ischemia with NOCAD [37].

A male predominance was also observed in the OCAD group, corroborating epidemiological trends indicating that men under 60 have a higher CAD prevalence [38]. This gender disparity is partly attributed to the cardioprotective effects of estrogen in premenopausal women. However, when traditional risk factors are adjusted for, the gender-based risk tends to equalize [35,38,39]. Thus, the observed male predominance in our cohort likely reflects both inherent biological risk and a greater accumulation of modifiable risk factors among men.

### 4.2. Glycemic Status and Insulin Dynamics

FBG levels were significantly elevated in the OCAD group, whereas FI levels, although higher in the NOCAD group, did not achieve statistical significance. The QUICKI did not differ markedly between the groups. The combination of elevated FBG, similar QUICKI values, and slightly lower insulin levels in the OCAD group suggests a distinctive metabolic profile potentially indicative of impaired insulin secretion rather than resistance. Previous studies have reported that coronary artery disease can be associated with impaired β-cell secretory capacity independent of insulin sensitivity [26,40]. This concept aligns with recent insights highlighting metabolic and electrophysiologic interplay between β-cell dysfunction and cardiovascular risk [41,42].

The observed metabolic profile aligns with characteristics of type 1 diabetes or insulin-dependent diabetes mellitus, where impaired pancreatic insulin release plays a central role. Prior research demonstrates that even pediatric IDDM patients can develop early atherosclerotic changes [43], with long-term follow-up studies linking IDDM to elevated CAD-related mortality, particularly after age 55 [44]. Given the older age profile of OCAD patients in this study, it is plausible that they represent a population with chronic IDDM and prolonged hyperglycemia, although this remains speculative and warrants further investigation.

Interestingly, despite elevated FBG levels, the mean QUICKI values in both groups remained above the threshold of 0.3, typically not associated with severe insulin resistance [45]. This observation further supports the hypothesis that the OCAD group may not conform to a classical type 2 diabetes mellitus (NIDDM) phenotype. Instead, they may represent individuals with long-standing IDDM or other comorbidities such as renal impairment or systemic inflammation, which contribute to the development of atherosclerosis through alternate metabolic pathways [45,46].

### 4.3. BMI and Metabolic Complexity

An unexpected finding in this study was the higher BMI observed in the NOCAD patients compared to those with OCAD. While elevated BMI is conventionally regarded as a risk factor for CAD [1,9], this inverse association has been documented in previous research. According to one report, obese diabetic patients had a lower prevalence of CAD after adjusting for potential confounders [17]. In the present study, the higher prevalence of diabetes in the OCAD group may partly explain their lower BMI, potentially reflecting weight loss associated with advanced disease progression or the catabolic effects of chronic illness [18]. Furthermore, systemic inflammation linked to late-stage atherosclerosis may induce a catabolic state, leading to involuntary weight reduction [9,19]. Age-related sarcopenia and unintentional weight loss, especially among older males who predominated the OCAD group, may also contribute to this pattern [20,21]. Long-term follow-up studies demonstrate that advanced CAD is often accompanied by systemic inflammation, malnutrition, and sarcopenic catabolism, contributing to lower BMI [47,48,49]. Thus, the lower BMI observed in OCAD patients likely reflects disease-related metabolic depletion rather than protection from atherosclerosis.

These observations highlight the importance of evaluating metabolic health across BMI categories. The relationships among glycemic control, lipid abnormalities, and body composition are complex and often non-linear [22]. Elevated BMI is commonly associated with insulin resistance, systemic inflammation, and disruptions in lipid metabolism [4,23]. These alterations often result in increased LDL-C and total cholesterol levels, along with reduced HDL-C, thereby elevating lipid risk ratios such as LDL-C/HDL-C and cholesterol/HDL-C [4]. However, these ratios may not solely reflect intrinsic lipid abnormalities; rather, they could represent downstream effects of broader obesity-related metabolic dysfunction [23].

In this study, HDL cholesterol was significantly higher in the NOCAD group compared to the OCAD group, supporting its well-established protective role in atherogenesis. Earlier studies, including the Framingham [50] and Quebec Cardiovascular [15] studies, have emphasized the predictive significance of HDL-C and LDL-C/HDL-C ratios. HDL-C plays a critical role in reverse cholesterol transport and exerts anti-inflammatory effects [8,51]. Accordingly, low HDL-C levels may indicate a transition toward a pro-atherogenic metabolic environment, even when overt dyslipidemia is not apparent. Thus, reduced HDL-C concentrations remain clinically meaningful as markers of cardiovascular risk [8,15,50,51]. This observation is reinforced by our finding of significantly higher HDL-C levels in the NOCAD group relative to the OCAD group.

LDL-C/HDL-C and cholesterol/HDL-C ratios were initially elevated in the OCAD group, consistent with a more atherogenic lipid profile. However, these associations were attenuated after adjusting for BMI, suggesting that the observed differences in lipid ratios may be driven in part by obesity-related metabolic disturbances rather than by primary dyslipidemia. Since elevated BMI is intertwined with insulin resistance, inflammatory signaling, and alterations in lipid processing, it can substantially influence lipid profile interpretation [20,21,22]. Therefore, although lipid ratios continue to serve as valuable cardiovascular risk indicators, their interpretation should be contextualized within the broader framework of adiposity and associated metabolic dysfunction [4,23].

### 4.4. BMI-Stratified Analysis of Metabolic Markers

BMI quartile analysis provided deeper insights into the metabolic variations between OCAD and NOCAD patients. With increasing BMI, FBG levels rose while QUICKI scores declined, signifying progressively impaired insulin sensitivity [45]. These findings reaffirm the association between elevated BMI and insulin resistance and further contextualize the earlier observed differences in glycemic control between OCAD and NOCAD patients.

OCAD patients, despite having lower BMI, presented with higher FBG but similar insulin sensitivity measures. This divergence may point to beta-cell dysfunction as the underlying driver of hyperglycemia in this subgroup. Conversely, those in higher BMI quartiles likely developed dysglycemia via insulin resistance mechanisms. Hence, BMI-stratified analysis not only clarifies the observed metabolic heterogeneity but also emphasizes the necessity for tailored risk assessments based on individual metabolic phenotypes.

Triglyceride levels increased with BMI, consistent with adiposity-driven lipid dysregulation. However, HDL-C and lipid ratios showed a more complex pattern: both peaked in mid-BMI quartiles before declining in the highest quartile. These non-linear trends suggest that lipid abnormalities may not linearly track with BMI and further support the hypothesis that elevated lipid ratios in OCAD patients reflect underlying metabolic conditions rather than intrinsic lipid disturbances. The loss of statistical significance in lipid ratio differences post-BMI adjustment strengthens this view, reinforcing the need to interpret such markers in the context of body composition and metabolic health.

### 4.5. Predictors of OCAD in Regression Analysis

In logistic regression analysis assessing predictors of OCAD, only a subset of variables, including age, male gender, and FBG, retained statistical significance. This outcome may be partially attributable to collinearity among interrelated factors such as age, lipid levels, and glycemic markers, which can obscure individual effects in multivariable models.

Additionally, pharmacological management likely influenced these results. Many patients undergoing ECA are treated with antihypertensive, lipid-lowering, or antidiabetic medications, which can normalize risk markers and reduce detectable associations in observational analyses [32,52].

Selection bias is another consideration, as referral for coronary angiography often occurs in patients with established risk factors, resulting in somewhat homogeneous risk profiles between OCAD and NOCAD groups. This homogeneity attenuates measurable differences and may explain the limited predictive power of some variables.

Nonetheless, the persistence of age, gender, and FBG level as significant predictors reinforces their fundamental role in CAD pathogenesis and supports their inclusion in clinical risk stratification frameworks.

### 4.6. Study Appraisal, Implications, and Future Directions

This study provides important insights into the metabolic and glycemic profiles of patients undergoing ECA. While the findings highlight significant associations between insulin resistance, lipid indices, and the presence of OCAD, certain methodological considerations must be acknowledged to guide and refine future research in this field.

First, the cross-sectional design, although suitable for identifying associations, does not allow for the determination of causal relationships. To establish temporality and better define the natural progression of metabolic dysfunction in relation to CAD, future investigations should adopt prospective, longitudinal cohort designs. This would clarify whether impaired insulin sensitivity contributes to the development of atherosclerosis or arises as a result of coronary disease-related inflammation and metabolic stress [53,54].

Second, although the sample size was adequate for initial exploratory analysis, expanding the cohort in future multicenter studies would enhance generalizability and statistical power. This is particularly crucial for regions like Sudan and other sub-Saharan African countries, where data on metabolic-cardiovascular interactions remain limited [6]. Broader sampling would also help refine risk stratification models tailored to the specific demographic, genetic, and socioeconomic characteristics of these populations.

Third, the data on specific pharmacotherapies were not systematically recorded in this study. However, since all participants received care under comparable tertiary-center protocols, both OCAD and NOCAD groups were likely exposed to similar standard treatments (statins, antihypertensives, and antidiabetic agents), thereby reducing (but not eliminating) the potential for confounding related to medication use.

Insulin resistance in the current study was assessed using a surrogate index, namely QUICKI. This tool is widely accepted in epidemiological and clinical settings due to its practicality and low cost. However, it remains an indirect measure. Employing gold-standard methodologies such as the hyperinsulinemic-euglycemic clamp in select future cohorts would provide more precise and physiologically informative assessments, though logistical and financial feasibility must be considered [6].

The implications of our study findings suggest several practical recommendations. First, insulin resistance markers such as QUICKI and FI should be considered in cardiovascular risk assessments, especially in settings where metabolic syndrome is increasingly prevalent [3]. These surrogate markers can help identify at-risk individuals who may not meet criteria for overt diabetes but still carry significant cardiometabolic risk [11].

Second, early lifestyle intervention is critical. Clinicians should promote dietary modifications, physical activity, and weight management strategies for patients with metabolic abnormalities, even in the absence of clinical diabetes [16]. These preventive measures have the potential to improve insulin sensitivity and reduce the burden of atherosclerotic disease [55].

Third, healthcare systems, particularly in low- and middle-income countries, should prioritize screening for metabolic derangements in primary care and cardiology clinics [56]. Men in midlife and individuals with a family history of metabolic or cardiovascular disorders may benefit most from early detection programs [57].

Fourth, future studies should integrate identified predictors into a locally validated risk-scoring algorithm to estimate the probability of >50% stenosis among symptomatic patients, potentially guiding non-invasive triage for angiography.

Region-specific guidelines that account for both traditional and emerging metabolic risk factors are urgently needed. These protocols must be contextually relevant and based on local epidemiological data [6,58]. Collaborative efforts among researchers, clinicians, and public health stakeholders are essential to develop and implement such guidelines effectively [5].

Looking forward, several avenues of research warrant attention. Longitudinal studies should investigate the natural history of insulin resistance and dyslipidemia in the development of CAD, with an emphasis on transitions from normoglycemia to prediabetes and diabetes [4,22,45,51]. Population-based studies involving diverse ethnic and socioeconomic groups can improve the external validity of metabolic risk thresholds [1,23,56].

## 5. Conclusions

This study highlights the complex interplay between demographic factors, glycemic control, insulin dynamics, and lipid metabolism in the context of OCAD. The findings affirm the critical role of advancing age, male gender, and elevated FBG as significant predictors of OCAD, reflecting well-established cardiovascular risk paradigms. Notably, the observed metabolic heterogeneity, characterized by impaired insulin secretion rather than classic insulin resistance and the nuanced relationship between BMI and lipid profiles, highlights the need for individualized risk assessment beyond conventional markers.

## Figures and Tables

**Table 1:** Characteristics of the studied groups.

	Non-Obstructive CAD (NOCAD, N = 66) N (%) Mean ± SD Median (Q1–Q3)	Obstructive CAD (OCAD, N = 72) N (%) Mean ± SD Median (Q1–Q3)	*p*
**Age (Years)**	54.85 ± 15.38	59.44 ± 10.38	0.044
**Males**	33 (50.0%)	58 (80.6%)	<0.001
**BMI (kg/m^2^)**	28.32 ± 4.97	26.58 ± 4.70	0.044
**MABP (mmHg)**	95.00 (87.00–105.67)	92.50 (83.42–106.67)	0.504
**Hypertension**	30 (45.5%)	34 (47.2%)	0.835
**Diabetes Mellitus**	19 (28.5%)	36 (50.0%)	0.011
**Smoking**	29 (43.9%)	38 (55.1%)	0.196

**Table 2:** Distribution of glycemic and lipid profile measurements among the studied groups.

	Non-Obstructive CAD (NOCAD, N = 66) N (%) Mean ± SD Median (Q1–Q3)	Obstructive CAD (OCAD, N = 72) N (%) Mean ± SD Median (Q1–Q3)	*p*	
			Non-Adjusted for BMI	Adjusted for BMI
**FBG (mg/dL)**	99.00 (86.75–123.50)	115.00 (94.00–172.00)	0.017	0.026
**FI (μIU/mL)**	16.05 (8.30–30.58)	11.40 (7.45–22.35)	0.055	0.169
**QUICKI**	0.31 ± 0.04	0.31 ± 0.03	0.800	0.783
**Triglycerides (mg/dL)**	87.50 (71.75–127.50)	91.00 (65.00–126.00)	0.651	0.829
**Cholesterol (mg/dL)**	145.50 (125.25–175.25)	145.00 (115.00–173.00)	0.364	0.792
**LDL-C (mg/dL)**	85.39 ± 31.71	86.87 ± 31.18	0.790	0.545
**HDL-C (mg/dL)**	40.82 ± 8.26	36.36 ± 9.80	0.006	0.037
**Cholesterol/HDL-C**	3.58 (3.17–4.29)	4.00 (3.40–4.63)	0.084	0.100
**LDL-C/HDL-C**	1.94 (1.71–2.56)	2.29 (1.75–3.02)	0.031	0.058

**Table 3:** Binary Logistic Regression Predicting Obstructive CAD.

Predictor	B	SE	Wald χ^2^	OR (95% CI)	*p*
**Age (years)**	0.065	0.022	8.580	1.067 (1.022–1.114)	0.003
**Gender (Male)**	1.366	0.498	7.517	3.921 (1.476–10.413)	0.006
**FBG (mg/dL)**	0.012	0.005	6.106	1.013 (1.003–1.023)	0.013
**FI (µIU/mL)**	−0.016	0.009	3.016	0.984 (0.967–1.002)	0.082

B = regression coefficient; SE = standard error; OR = odds ratio; CI = confidence interval.

## Data Availability

The data generated or analyzed in this study can be obtained from the corresponding author upon reasonable request.
